# Ultrasound-assisted process to improve proteins recovery from industrial canola and soybean byproducts

**DOI:** 10.1007/s13197-024-06108-8

**Published:** 2024-10-14

**Authors:** Demelza Nayelli Villalón-López, Laura Patricia Martínez-Padilla

**Affiliations:** Laboratorio de Propiedades Reológicas y Funcionales en Alimentos, Facultad de Estudios Superiores Cuautitlán, Universidad Nacional Autónoma de México, Av Primero de Mayo s/n, 54740 Cuautitlán Izcalli, Edo de Mexico Mexico

**Keywords:** Canola, Soybean, High-power ultrasound, Protein isolates, Phenolic compounds, Byproducts

## Abstract

An ultrasound-assisted process is proposed to obtain protein isolates from canola and soybean byproducts. A laboratory-scale probe system (24 kHz, 400W) was used for protein extraction and six levels of energy density were analyzed. Different mixtures of solvents (water, ethanol, methanol, acetone) and temperatures were tested for removing phenolic compounds, and the best results were obtained with ethanol-water (8:2 v/v) and energy density of 104 kJ/L, which minimize protein losses at that step. After extraction of phenolic compounds, samples were alkalinized (pH 12 or 9.5), subjected to ultrasound treatment and the protein precipitated at the isoelectric pH. The highest yield on protein extraction was obtained with 1:20 meal: NaOH solution ratio, applying 150 kJ/L at constant temperature (30 °C), increasing the protein recovery, 72.5% for canola meal and 37.5% for soybean meal, compared to the conventional method. The solubility of both isolates obtained with ultrasound improved ~ 50% at a pH close to neutral and basic, with an increase in free sulfhydryl groups, without significant effects of ultrasound treatment on the subunit fractions of the proteins. These results provide alternatives to develop protein isolates from an undervalued by-product, with better techno-functional properties that can be employed in the food industry.

**Supplementary Information:**

The online version contains supplementary material available at 10.1007/s13197-024-06108-8.

## Introduction

Canola and soybean meals, byproducts of oil pressing, are frequently used for consumption by poultry, pigs, aquaculture, and livestock. Meals have been used as a substitute for petroleum products such as adhesives, coatings and printing inks, lubricants, biodiesel and plastics (Brentin 2014; Fetzer et al. 2020). Canola, a variant of *Brassica napus* and *Brassica rapa* species (rapeseed), is an oilseed that is among the first crops exploited and is a valuable source of high-quality protein for human consumption. Additionally, studies have recognized rapeseed meal as a possible alternative protein source due to its well-balanced amino acid composition and technological functionalities (Yagoub et al. 2017) even it is analogous to soy protein in nutritional value and comprises more sulfur containing amino acids than other leguminous species. In addition, canola byproducts of oil-producing countries, represent a source of a viable food ingredient.

The utilization of protein from plant sources such as the Brassicaceae family (*i.e.,* canola, rapeseed, brown and white mustard) is limited by the presence of glucosinolates, phytates, hulls, and phenolic compounds. Particularly for rapeseed, phenolic acids and condensed tannins are the main polyphenols, which can be compared to canola due to their common origin. The high content contributes to a bitter taste and astringency, and forms complexes with proteins. Phenolic compounds are hydrophilic, so the use of polar solvents or a mix of them, temperature, appropriate particle size, and solvent-solid ratio, can maximize their extraction (Nandasiri et al. 2019).

Moreover, some problems arise during the oil extraction process such as undesired reactions among proteins and between proteins and phenols (Xu and Diosady 2000) and cell disruption, which have a negative influence on the physicochemical properties of the remaining proteins and impact extraction yields. Processing of meals may also influence the amino acid pattern and digestibility, leading to different quality scores. Besides, during the conventional alkaline method of protein isolation, some of the protein remains in the precipitate (van den Berg et al. 2022).

Ultrasound technology for assisting the extraction process was proposed since showed potential from the laboratory to industrial scale, due to the relatively inexpensive equipment, simple procedure and high efficiency (Preece et al. 2017). The cavitation effect is the key factor due to the shear forces and the shock waves generated. The formation, growth, and implosive collapse of microbubbles inside the liquid, and pressure at the zone of bubble implosion facilitate fragmentation of particles, disrupt aggregates, increase the permeability of the plant tissue, and release intracellular material and heat generation. However, prolonged ultrasound treatment (UST), providing high ultrasound energy density (${E}_{density}$), may lead to excessive cavitation, and aggregation, resulting in a reduction in mass transfer and decreased protein extraction efficiency (Suchintita-Das et al. 2022).

This study aims to establish the process steps and best conditions for the extraction proteins for soy and canola byproducts. Chemical composition was evaluated, and the molecular size distribution of the proteins was determined before and after UST at 150 kJ/L. First, different solvents and temperatures were evaluated to remove phenolic compounds. Second, different ${E}_{density}$ at constant temperature were assessed for protein extraction. The extraction protein yield was calculated at 150 kJ/L and a comparison was established with the conventional method (CM). An additional ${E}_{density}$ (150 or 185 kJ/L) was applied to isolates, and the solubility and free sulfhydryl groups were measured at different pH values, the latter related to the improvement of protein functionality.

## Material and methods

### Materials

Canola meal was obtained from an oil factory Industrial Aceitera SA de CV (Mexico) and soybean meal from Productos Avícolas el Calvario SA de CV (Mexico). All the chemicals used in the study were of analytical reagent grade.

### Chemical composition

Soybean meal was dried at 50 °C overnight and stored. The moisture content was determined by thermogravimetry (90 °C, A60, Ohaus MB45, Switzerland). The lipid content was determined in Soxhlet apparatus with hexane, and desolventizing at T_room_ overnight. Crude ash (923.03, AOAC, 2012) and total protein content were determined, the latter using the micro Kjeldahl method (60.52, 945.39 AOAC, 2012) (N_2_ × 6.25), with 150 mg of the dried meal and 43 mg of protein isolate. For carbohydrate content, the anthrone method was used, meal (100 mg) was hydrolyzed by 3 mL HCl (2.5N) at 10 °C overnight. It was neutralized by Na_2_CO_3_ until it stopped fizzing; the extracts were gauged at 25 mL (volumetric flask) and centrifuged (3720 g, 10 min, Universal 320, Hettich, Germany). The extracts were measured at 620 nm (Thermo Scientific UV‒vis, USA), using glucose as standard.

Protein individual fractions were determined by Bradford method with modifications (20 $\mu$L sample/1 mL Bradford reagent), using BSA as standard. Meal (300 mg) was extracted consecutively with 1.5 mL of each solvent: distilled water (albumin), 5.0 M NaCl (globulin), absolute ethanol (prolamin), 0.2 M phosphate buffer at pH 8 (glutelin). Each extract was vortexed (Maximix I, Thermo Scientific) for 25 min (T_room_) and centrifuged (7905 g, 6 min).

Protein molecular size distribution was determined by SDS-PAGE technique. Samples with a 1:3 v/v ratio of Laemli buffer, 65.8 mM-Tris–HCl pH 6.8 buffer containing 2.1% w/w SDS, 26.3%, glycerol, 0.01% w/v bromophenol blue for nonreduced conditions. For reduced conditions, samples were dissolved in Laemli buffer, with the addition of 5% (v/v) 0.335 M of $\beta$-mercaptoethanol. Afterward, the samples were heated at 90 °C for 6 min and vortexed; 15 $\mu$L of each sample was loaded into a hand-casted gel (5%, stacking gel;10%, running gel) using a protein standard (10–250 kDa). The electrophoresis was performed at 80 V for stacking voltage (25 min) and 120 V for separation voltage (~ 2 h). Gels were stained using 0.125% Coomassie blue R-250, 40% methanol and 7% acetic acid. For destained 20% of methanol and 10% acetic acid were used.

To determine the total phenolic content, the extract was dried, transferred and filled with water to 100 mL in a volumetric flask, followed by centrifugation (7440 g, 15 min). The total phenolic content was determined by the Folin-Ciocalteu method with gallic acid as standard. The diluted extract (300 $\mu$L) was added to 3 mL of distilled water and 200 $\mu$L of the reagent was added. After 6 min, 600 $\mu$L of 20% Na_2_CO_3_ was added, and the contents were vortexed and incubated, 120 min. The color developed was measured at 765 nm. The results were expressed as milligrams of gallic acid equivalents (GAE)/ 100 g meal.

### Extraction procedures

**Fig. 1:**
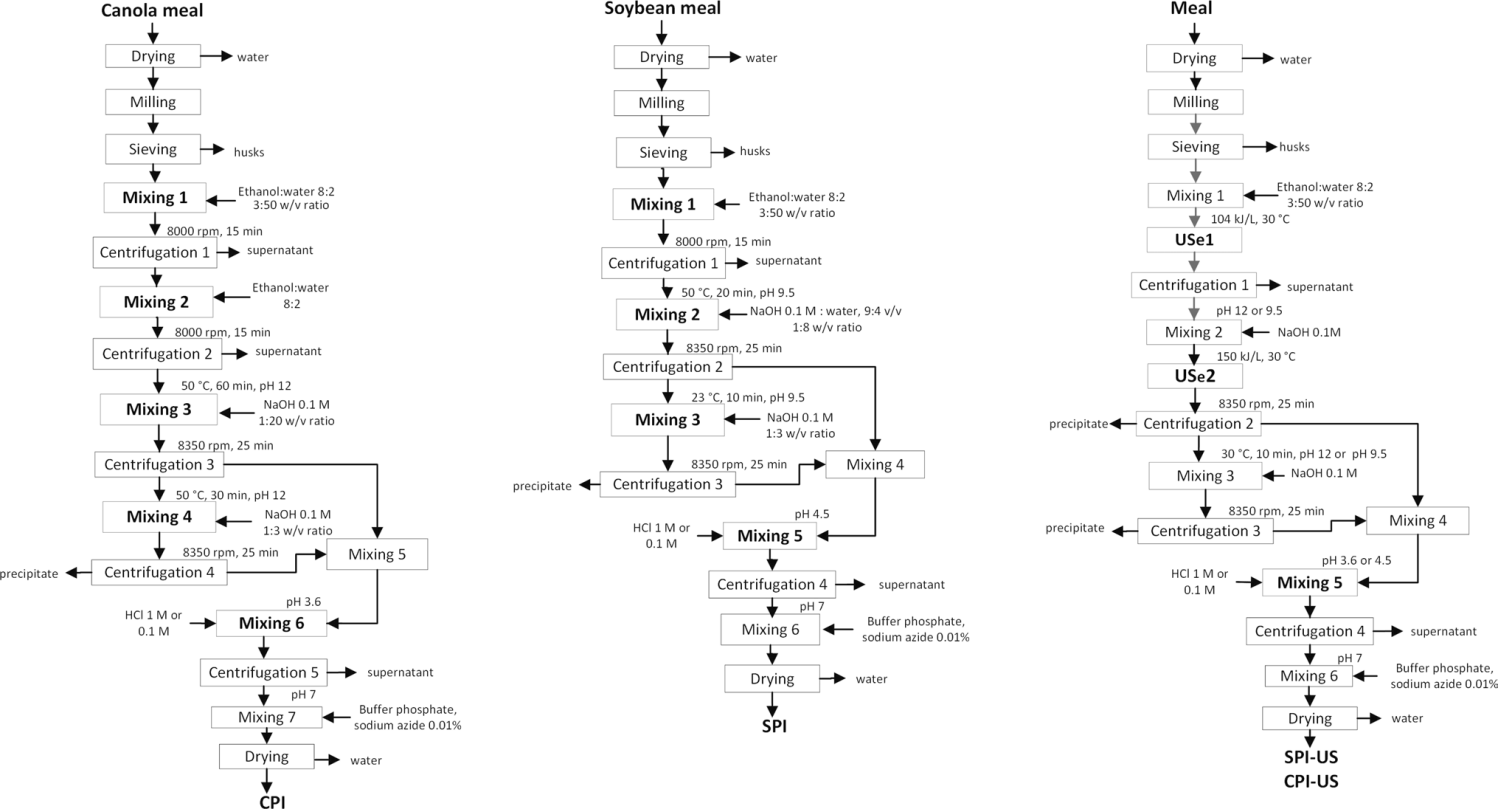
Steps carried out in obtaining canola (CPI) and soybean (SPI) protein isolates by conventional method from industrial byproducts. The ultrasound-assisted process is also shown, including the USe1 (104 kJ/L = 123.3 kJ/kg) and USe2 (150 kJ/L = 148.8 kJ/kg) interventions

Schematic diagrams of the process applied to obtain each of the isolates, as well as the sonicated isolates, are shown in Fig. 1. The meal was dried, ground, and passed through a 65 mesh (210 $\mu$m) sieve to remove the husks. Various mixing and centrifugation operations were performed, first with solvents to remove phenolic compounds and others to alkalinize the samples and separate by isoelectric precipitation to obtain the canola (CPI) and soy proteins (SPI) isolates. The samples that were treated with UST, both for the extraction of phenolic compounds (USe1) and for the extraction of proteins (USe2), are shown in the third diagram, where the samples were dried to obtain sonicated isolates (SPI-US, CPI-US). Meals were kept in plastic containers (T_room_) for next steps.

For phenolic compound extraction, acetone:water (7:3 v/v), ethanol:water (8:2 v/v), methanol:ethanol:water (7:7:6 v/v), and ethanol:water (16:3 v/v, 50 °C) were tested taking as reference studies on hemp, flax and canola seed cakes. The samples (3 g) were mixed with the solvent (50 mL), and stirred at 700 rpm (magnetic stirrer, Cimarec, Barnstead Thermolyne, Malaysia) for 60 min (T_room_), except that which was mixed at 50 °C (20 min). The samples were centrifuged (7440 g, 15 min), the precipitate washed with the same solvent, 50 °C, 15 min, and centrifuged (7440 g, 15 min). The supernatant was evaporated to dryness, then dissolved with distilled water (500 $\mu$L) to determine the soluble protein (Bradford method). The precipitate was used for the next step.

For canola protein extraction, the method of Qu et al. (2018) was used with modifications. Meal was dispersed (magnetic stirrer, 700 rpm) with 0.1 M NaOH (1:20 w/v ratio), at pH 12 ± 0.05, adjusted with 6 N NaOH using a digital pH-meter (Oakton pH 700, Singapore), 60 min, 50 °C. A second extraction with 0.1 M NaOH at same ratio and pH was done (30 min, 50 °C). Both extracts were centrifuged (7766 g, 25 min). Supernatants were pooled and precipitated at pH 3.6 ± 0.05, using 3 N and 0.1 N HCl, and centrifuged (same conditions). The protein was adjusted to pH 7 ± 0.05 with phosphate buffer, containing 0.01% sodium azide, dried (50 °C, 4 h) and left overnight (T_room_).

For soybean protein extraction, the method of Jiang et al. (2012) was used with some modifications. Meal was dispersed (magnetic stirrer, 700 rpm) with 0.1 M NaOH: H_2_O (9:4 v/v ratio) solution at a 1:8 ratio (w/v) at pH 9.5 ± 0.05, adjusted with 6 N NaOH, 20 min, 50 °C, centrifuged (7766 g, 25 min). Precipitates were washed with the same NaOH: H_2_O solution, 1:3 w/v, 10 min (T_room_) and centrifuged (same conditions). Both extracts were pooled and precipitated at pH 4.5 ± 0.05 with 3 N and 0.1 N HCl and centrifuged (same conditions). The protein was adjusted to pH 7 with phosphate buffer, containing 0.01% sodium azide, dried (50 °C, 4 h) and left overnight (T_room_). The protein recovery yield ($PRY$) was calculated as:

1 $$PRY (\%)=\frac{{Precipitated\; protein \,\left(g\right)}}{Protein\; in \;meal\; \left(g\right)}\times 100$$

### Ultrasound-assisted methods

Preliminary studies were conducted to choose the sonotrode, depth immersion, ${E}_{density}$ and pulse. A Hielscher UP400St (Germany) ultrasonic processor system was used, with operating frequency (24 kHz) and nominal power (400 W). A titanium sonotrode S24d22D (22 mm diameter, submerged depth 45 mm) was selected. The pulse was maintained constant at 30% (ON 2 s / OFF 4 s cycle). The temperature was controlled by an ice bath or boiling water bath. Different levels of ${E}_{density}$ (kJ/L) were obtained by varying the amplitude to 90% (41.4 $\mu$m) or 100% (46 $\mu$m), it was determined by the method described by Rahman et al. (2021) and was calculated as:

2 $${E}_{density}= \frac{P}{V}$$

where $P$ is the ultrasound net power (W s), and $V$ is the volume of sample (L).

For phenolic compound extraction, based on previous experiments and maximum yields observed, a constant ${E}_{density}\hspace{0.17em}$= 104 kJ/L (90% amplitude, USe1) was proposed to evaluate the effect of UST on the different solvents studied. The temperature was kept at 30 °C. A constant volume of 150 mL of sample was maintained with the intention of having a constant relationship between the volume of the sonotrode tip and the volume of the sample.

For protein extraction, the ${E}_{density}$ to obtain the protein isolates was varied in six levels: 78.5, 110, and 150 kJ/L (100% amplitude) and 61.5, 80.5, and 132 (90% amplitude). The processing time was less than 20 min for 150 mL samples. The $PRY$ was calculated. The samples with low phenolic compounds removed with ethanol: water (8:2 v/v, 104 kJ/L, 123.3 kJ/kg) was used for next step.

To obtain CPI an ${E}_{density}$ of 150 kJ/L (148.8 kJ/kg), USe2, was selected: First. the sample was mixed with 0.1 M NaOH, 1:20 ratio (w/v) containing reducing agents, 0.5% Na_2_SO_3_ and 0.05 N NaCl and 0.05 N CaCl_2_ to break the remaining phenolic-protein complex and with low phytate, respectively, adjusted to pH 12 ± 0.05 with 6 N NaOH, as proposed by Tan et al. (2011a) and centrifuged (7766 g, 25 min). Precipitates were washed with 0.1 M NaOH (1:7 w/v) and centrifuged (same conditions). The supernatants were collected and adjusted to pH 3.6 with 3 N and 0.1 HCl and centrifuged (same conditions). The protein was, adjusted to pH 7.0, dried (50 °C, 4 h) and left overnight (T_room_).

To obtain SPI the sample was mixed with 0.1 M NaOH:H_2_O (9:4 v/v ratio) solution at a 2:15 ratio (w/v) adjusted with 6 N NaOH at pH 9.5 ± 0.05. The same reducing agents and the same UST (USe2) were applied. The dispersion was then centrifuged (7766 g, 25 min). Precipitates were washed with NaOH:H_2_O solution (1:7 w/v) and centrifuged (same conditions). The supernatant was adjusted to pH 4.5 with 3 N and 0.1 N HCl and centrifuged (same conditions). The protein was adjusted to pH 7.0 and dried (50 °C, 4 h) and left overnight (T_room_).

### Isolates solubility and free sulfhydryl groups

The protein solubility was determined as a function of pH, the isolates were dispersing in 0.2 M of buffer phosphate to obtain a 50 mg/mL at different pH and vortexed 5 min every 15 min after 60 min, and centrifugated (3720 g, 10 min, T_room_). Protein solubility ($PS$) was calculated as:

3 $$PS\left(\%\right)=\frac{{Supernatant\,protein\,concentration}_{\left(\frac{mg}{mL}\right)}\times {Volume}_{\left(mL\,of\,sample\right)}}{{sample weight}_{\left(mg\right)}\times \frac{{Sample\,protein\,concentration}_{\left(\%\right)}}{100}}\times 100$$

Furthermore, to evaluate the effect of applying UST to the isolates, the protein was dispersed (7 mg/mL) in 0.2 M buffer phosphate at different pH values with magnetic stirring (5 min), treated with additional UST at two ${E}_{density}$, 185 and 150 kJ/L (90% amplitude), 30% pulse, 30 °C, and centrifuged (3720 g, 10 min). The protein content was analyzed according to the Bradford method.

To determine the total free sulfhydryl groups, meal and isolates were dissolved in the reaction buffer (0.1 M sodium phosphate, pH 8.2 containing 1 mM EDTA) at 8 mg/mL and 7 mg/mL respectively, and vortexed (10 min). Additionally, different pH values were probed, dispersing the protein in 0.2 M of buffer phosphate. Also, the effect of two ${E}_{density}$ was assessed on the isolates, at 185 and 150 kJ/L, 30 °C. Ellman’s reagent was prepared by dissolving 4 mg of DTNB in 1 mL of reaction buffer, using L-cysteine as standard. Then, 340 $\mu$L of reaction buffer was mixed with 880 $\mu$L of protein solution, and 80 $\mu$L of Ellman’s reagent. After placing it in the dark for 15 min at (T_room_), the absorbance was read at 412 nm, using the reaction buffer with Ellman’s reagent as the blank control. Free sulfhydryl groups ($SH$) were calculated as follows.

4 $$SH=\frac{73.53\times {A}_{412}\times FD}{{C}_{IP}}$$

where ${A}_{412}$ is the absorbance at 412 nm; $FD$, the dilution factor; and ${C}_{IP}$, the concentration of the isolate samples (mg/mL). The supernatant obtained after protein precipitation was also analyzed without dilution.

### Statistical analysis

All analyses were performed in triplicate. Results reported as the mean ± standard deviation. One-way ANOVA (Tukey test) was applied to compare the means of the replicates (*p* < 0.05). Minitab 17 for Windows (Pennsylvania, USA) was used.

## Results and discussion

### Composition of meals and protein fractions

The chemical composition of meals was determined after removing the husks, associated with most glucosinolate compounds (Ghodsvali et al. 2005). The total protein and protein fractions for canola and soybean meals and isolates are shown in Table 1. The process of oil extraction and the last step to obtain the meal affect the protein content, where glutenin content was the protein fraction in the highest proportion, followed by albumins, globulins, and prolamins for both meals.

**Table 1:** Chemical composition and fraction protein of the studied byproducts and the canola (CPI-US) and soy (SPI-US) protein isolates obtained by ultrasound assisted extraction

	Canola meal	Soy meal	CPI-US	SPI-US
Moisture (%)	1.96 $\pm$ 0.08	5.18 $\pm$ 0.14	4.71 $\pm$ 0.2	4.05 $\pm$ 0.01
Protein (%)	48.4 $\pm$ 3.09	52.7 $\pm$ 2.8	78.5 $\pm$ 1.13	87.9 $\pm$ 3.14
Albumins (%)	13.74 $\pm$ 0.33^FG^	13.67 $\pm$ 0.23^FG^	7.00 $\pm$ 0.58^I^	13.18 $\pm$ 0.55^G^
Globulins (%)	10.18 $\pm$ 0.73^H^	14.14 $\pm$ 0.29^F^	30.79 $\pm$ 0.06^B^	5.16 $\pm$ 0.19^ J^
Prolamins (%)	6.99 $\pm$ 0.28^I^	6.86 $\pm$ 0.21^I^	16.51 $\pm$ 0.17^E^	34.70 $\pm$ 0.09^A^
Glutenins (%)	18.33 $\pm$ 0.42^C^	17.05 $\pm$ 0.47^DE^	17.84 $\pm$ 0.07^CD^	30.20 $\pm$ 0.98^B^
Lipids (%)	1.67 $\pm$ 0.55	2.23 $\pm$ 0.14	–	–
Ash (%)	5.99 $\pm$ 0.08	6.49 $\pm$ 0.02	–	–
Crude fiber (%)	10.6 $\pm$ 0.48	4.72 $\pm$ 0.31	–	–
Soluble carbohydrates (%)	15.3 $\pm$ 0.41	18.9 $\pm$ 0.79	1.09 $\pm$ 0.08	2.00 $\pm$ 0.15
Phenolic compounds (mg GAE/100 g)	4029.1 ± 16.0*	224.81 $\pm$ 5.79*	357.3 $\pm$ 8.34**	95.2 $\pm$ 0.49**

Different letters (A, B, C) show statistically significant differences between samples with a 95% confidence level

Mean ± standard deviation

^*^Ultrasound extracted with ethanol:water 8:2

^**^Extracted with ethanol:water 8:2

Electrophoresis showed differences in the protein profile of both isolates with UST and CM. The profile proteins in reducing and nonreducing conditions are shown in Fig. S1. The SDS-PAGE pattern of soybean protein involved two major regions of globulin, 7S (*β*-conglycinin) at ∼40.5–14.35 kDa and 11S (glycinin) at ∼67.4–41.35 kDa proteins. Detectable bands at ∼81.70–51.92 kDa can be identified as *α* and *β* subunits of 7S globulin, and another detectable band appears at ∼59.65 kDa which can be β-amylase and ∼93.35 kDa as lipoxygenase. Comparing UST and untreated soybean protein, the first did not produce changes in the protein electrophoretic patterns or modify the protein profiles, and the results are in accordance with others under different sonication conditions (Chen et al. 2011).

Canola protein patterns at pH 7.4, 8, and 10 also did not reveal differences in the protein profiles and were mainly composed of the 12S globulin (cruciferin) and 2S albumin (napin) forms. When CPI was treated at basic pH and UST, the subunits of cruciferin could be modified due to the disulfide bridges in the $\alpha$ and $\beta$ subunits. Moreover, during alkaline extraction, the addition of NaCl could more easily extract cruciferin protein, showing more prominent bands of ∼48.5–37.6 kDa, corresponding fairly with the monomeric 12S globulin (analogous to glycinin) subunits that were reported in the literature (Dong et al. 2011; Jia et al. 2021); additionally, subunits ∼29.2–17.8 kDa were found. Under reducing conditions, the first band almost disappeared, indicating the presence of disulfide bonds. Most of the visible peptide bands were in the ∼31.2–8.3 kDa range, where napin (strong basic protein) was identified in the range of ∼11.2–16.8 kDa, composed of two polypeptide chains, detected at 4.18 kDa small subunit and a large 9.78 kDa subunit, stabilized primarily by S‒S bonds; in addition, cruciferin separated into *α*-polypeptides (26.97–33.74 kDa) and *β*-polypeptides (18.69–20.70 kDa), which is in agreement with the values previously reported (Dong et al. 2011).

### Phenolic compounds extraction

**Fig. 2:**
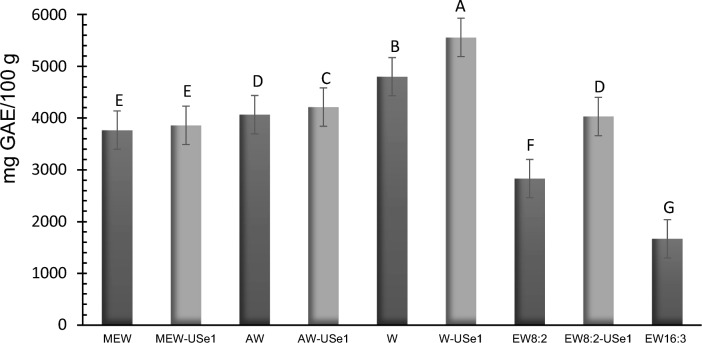
Phenolic compounds evaluated as gallic acid equivalent (GAE) of canola meal extracted with different mixture of solvents by conventional method and by ultrasound-assisted extraction (104 kJ/L, 30 °C, USe1). MEW, methanol:ethanol:water (7:7:6 v/v); AW, acetone:water (7:3 v/v); W, water; EW8:2, ethanol:water (8:2 v/v), ∼ 21 °C; EW16:3, ethanol:water (16:3 v/v) at 50 °C

The phenolic compounds extracted from canola meal by water and different mixtures of solvents, with 104 kJ/L, with ethanol:water 8:2 v/v (USe1) or by CM are shown in Fig. 2 and Table S1. The highest extraction was obtained by UST with water at 30 °C (5558.5 mg GAE/100 g meal) followed by water at T_room_ (4799.9 mg GAE/100 g meal). Similar phenolic compounds were extracted in sonicated samples with acetone: water (7:3 v/v, 126.06 kJ/kg) and ethanol: water (8:2 v/v) with UST (30 °C) (Table S1). The extraction with water affected the final protein content in the isolates due to the total soluble protein recovered in supernatants with UST (1.66 $\pm \hspace{0.17em}$0.014%) and using the CM (1.91 $\pm \hspace{0.17em}$0.83%), which decreased the yield in obtaining the protein isolate.

Using ternary and binary systems by CM, such as methanol:ethanol:water (114.07 kJ/kg) and acetone:water, 3766.7 to 4065.3 mg GAE/100 g meal of phenolic compounds were extracted. It has been found that the variability of genetic characteristics and environmental factors influence the amounts of phenolic compounds extracted, as well as, the solvent system and pretreatment of the oilseeds (*i.e.* industrial oil extraction) (Nandasiri et al. 2019). The phenolic compounds extractability assisted with UST (USe1) by methanol:ethanol:water and acetone:water can be compared to ethanol:water 8:2 v/v (4029.1 mg GAE/100 g meal) while by CM was lower extraction (2831.3 mg GAE/100 g meal) as shown in Fig. 2.

Two hydroalcoholic systems were also studied at different temperatures (50 °C and 30 °C), where the lowest recovery (1667.8 mg GAE/100 g meal) was attained using a higher concentration of ethanol (16:3 v/v) and higher temperature (50 °C). In some cases, high temperatures can disrupt the bonds between polyphenols and the protein matrix, enabling their faster release; but at 50 °C with UST, could increase the solvent vapor pressure that fills in the cavitation bubbles which subsequently collapse with minor strength reducing the impact of cavitation (Suchintita-Das et al. 2022). Moreover, using higher temperatures around 50 °C the net power generated by the sonicator decreased constantly compared to 30 °C, making the repeatability of the process difficult. In addition, an increase in processing time was observed. These results confirmed that the use of UST, 29 °C over T_room_ (*i.e*., 50 °C), did not improve the extraction of polyphenols. Consequently, for this study, ethanol:water extracts obtained by UST were chosen for the next step with a considerable increase in extraction of 29.73% compared to the CM with the same solvent. UST-extraction with water was discarded for affected the final protein content (yield) of isolates and compared with CM the extraction only increased 13.65%. The extractions of phenolic compounds by UST (104 kJ/L) at 30 °C had a processing time of $\sim \hspace{0.17em}$10–15 min, reducing it by 81.25% compared to the CM (∼ 60 min). Therefore, UST-extraction using ethanol:water (8:2) was selected and the intermediate product, was used for alkaline protein extraction for the next step.

The phenolic content was also measured in soybean meal, being 224.81 $\pm \hspace{0.17em}$5.79 mg GAE/100 g meal, 18 times lower when compared to canola meal which confirms that soybean does not contain phenolic compounds that may be viable to recover using an aqueous extraction. Overall, the total polyphenol content in meals varied depending on the method of extraction and solvents used.

The phenolic compounds in isolates obtained by UST-extraction are shown in Table 1 and Table S1. Generally, the residues of these compounds are slightly higher when using a mixture of ethanol:water compared to only water. These results confirm the usefulness of applying UST for significant extraction. On the other hand, phenolic compounds recovered from the meals could be used as bioactive ingredients with multifunctional features, and can be correlated with antioxidant capacity products, taking advantage of the fact that sinapine is the most abundant component in canola meal (∼ 80%) with potential antioxidant properties, followed by sinapic acid (hydrolyzed product of sinapine, 65 to 85%) and canolol (decarboxylated product) compounds with high antioxidant capacity (Nandasiri et al. 2019).

### Protein extraction and recovery

**Fig. 3:**
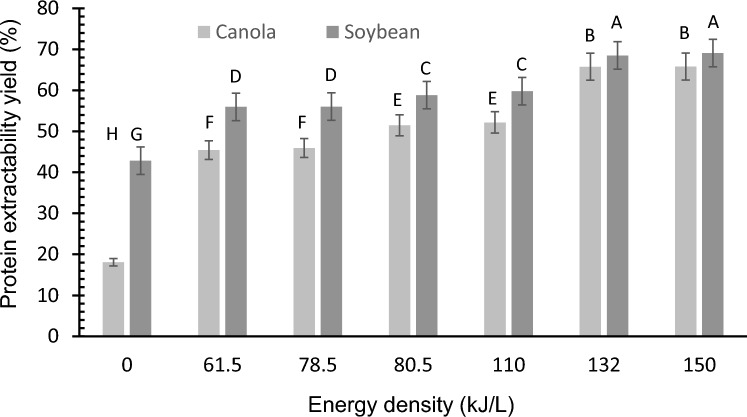
Protein extractability yield (30 °C) of canola and soybean meals applying various energy densities

Canola $PRY$ using the CM was 18.09% and for soybean meal was 42.84% (Fig. 3). The $PRY$ for canola meal obtained by the CM was lower than other defatted rapeseed meal used to obtain protein isolates that are close to 36% (Jia et al. 2021). However, Das-Purkayastha et al. (2014) obtained yields of 15.6% when rapeseed meal was treated with solvents and acidic pH to reduce phenolic compounds. For soybean meal and soybean flour, yields in a wide range depending on the extraction method have been reported*, i.e*. 65.66%, for extraction with ammonium hydroxide (Bello et al. 2023). The differences in protein recovery are mainly due to the heterogeneity of the nature of the byproducts with various components that can facilitate protein interactions through the conformation achieved by modifying the net charge and hydrophobicity of proteins affecting their extractability (Tan et al. 2011a).

When UST was applied during the protein extraction, $PRY$ increased proportionally to the ${E}_{density}$ delivered by the sonotrode (Fig. 3). However, significant differences were not observed between 61.5 and 78.5 kJ/L, 80.5 and 100 kJ/L, and 132 and 150 kJ/L, corresponding to 90% and 100% amplitude, respectively. The same proportional increase was observed for soybean meals (Rahman et al. 2021). In other studies, it was found that UST has a nonlinear increase with extractability at different sonication powers and times, that is, although there is an increase due to the US effect, the extracted protein reaches a maximum value independent of the power supplied, as reported for a water-soluble pea protein (Gao et al. 2022) and hemp seed (Yao et al. 2023).

The 1:20 meal:NaOH solution ratio showed a good extraction yield in this study, where canola $PRY$ with UST reached 65.76% at the highest ${E}_{density}$ studied (150 kJ/L, 30 °C). Yagoub et al. (2017) obtained a lower yield (~ 43%) in rapeseed protein (40% ultrasound power, 0.228 W/cm^2^). For soybean protein the $PRY$ reached 68.52% (150 kJ/L, 30 °C), these results are close to those reported by Rahman et al. (2021), 77.5% in defatted soybean flakes (720 kJ/L,15 min, pH 8.5, 60 °C) and 72.91% using 2.2 kW, 84 $\mu$m and 120 s (Karki et al. 2009). However, Das et al. (2023) for soybean meal obtained a maximum yield of 24.17%, and high protein purity (91.6%) by response surface methodology. The purity of soy and canola protein obtained by US-assisted extraction is shown in Table 1. The proteins extracted from these by-products were considered protein isolates, taking into account that in a study of seeds from various varieties of canola, isolates with a protein concentration of up to 73.23% have been reported (Aluko and McIntosh 2001).

Additionally, the supernatants were analyzed (Table S1), these have protein concentrations around 3–4%, so the type of extraction and the conditions of the raw material are key factors in extraction yield and purity. The precipitation of proteins at their isoelectric point seems to be specific to certain types of proteins, leaving some soluble proteins in the acidic supernatant that have different properties than those of isolated. Moreover, these supernatants can form Aquafaba-type foams.

The isolate proteins, provided different protein profiles (Table 1), where the globulin fractions were the ones with the highest proportion in CPI-US, followed by glutenins and prolamins, while in SPI-US the highest were prolamins, followed also by glutenins. Tan et al. (2011b) reported a maximum yield of glutenins, in samples of Australian canola meals prepared by a direct alkaline extraction method. When comparing the total protein content with the sum of each fraction, percentages lower than the content evaluated by the Kjeldahl method were observed except for canola meal. However, this difference was not greater than $\pm$ 2% in the meals and less than 9% in the isolates. The latter had lower protein content than those reported for isolates obtained with UST, 84.59% (Yagoub et al. 2017) and 79.63% (Dong et al. 2011) for rapeseed, and 94.3% for hexane-defatted soy flakes (Karki et al. 2009).

### Protein solubility as a function of pH

**Fig. 4:**
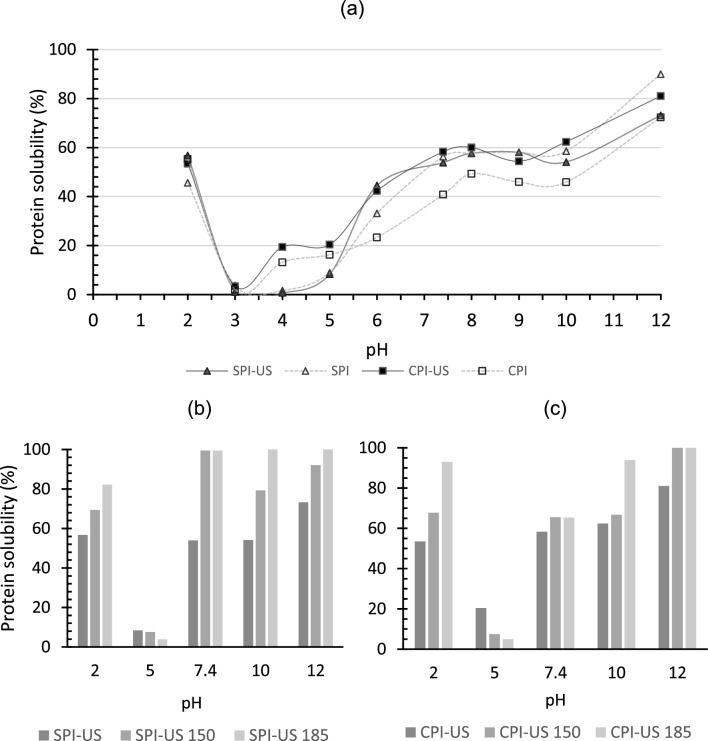
Solubility of canola (CPI) and soybean (SPI) protein isolates obtained by conventional method and by ultrasound-assisted extraction (US) (150 kJ/L, 100% ultrasound amplitude, USe2) (**a**). Changes in solubility of protein isolates obtained by ultrasound treatment. Soybean (SPI-US) (**b**) and Canola (CPI-US) (**c**) dispersed in phosphate buffer (0.2 M) and treated with additional UST, 185 or 150 kJ/L (CPI-US 185, SPI-US 185; CPI-US 150, SPI-US 150, respectively) are included

As an index of protein functionality, such as protein denaturation and aggregation, the most practical measure is protein solubility; the changes as a function of pH of CPI and SPI obtained by CM and UST-extraction are in Fig. 4a. Both isolates exhibit a typical U-shaped solubility. Using the CM, the lowest solubility of SPI was detected near the isoelectric pH, such as pH 3 and pH 4, and for CPI the lowest solubility was found at pH 3, which is also close to the isoelectric point (pH 3.6) of the protein. In the CM, at pH 2, the solubility of both isolates was similar as found at pH 7.4 to pH 10, $\sim$ 50%, however, the highest solubility was found at pH 12, 90.01%, and 72.35% for SPI and CPI, respectively. Similar results were reported for CPI obtained by CM, observing a maximum solubility of 74.32% at pH 12 (Flores-Jiménez et al. 2019).

In general, when the proteins were extracted with UST, an increase was observed in solubility, where the highest was also found at pH 12. However, SPI-US had a decrease in solubility at 23.0%, while CPI-US increased at 10.7%. The reduction in solubility in the SPI-US could be due to possible structural reorganization due to intermolecular interactions; even the possibility of nucleation of protein as a result of the higher ultrasonic frequencies as observed by Das et al. (2023) contrary to observations with CPI-US could suggest that sonication in this case can induce structural transformations, allowing strong interactions between water-protein molecules (Li et al. 2020).

The effect of an additional UST, 150 and 185 kJ/L, applied to the SPI-US and CPI-US at different pH values is shown in Fig. 4b and c where the highest solubility was observed in SPI-US 185 at pH 7.4, 10 and 12 nearly 100%, while CPI-US 185 also was (92–99%) at pH 2 and alkaline pH, 10–12. In contrast, a lower solubility was reported from CPI at pH 12 (78%), with 30 min of ultrasonic bath treatment (40 kHz, 130 W, 1 W/cm^2^) applied (Flores-Jiménez et al. 2019). The difference in the increase in the solubilities when an additional ${E}_{density}$ was applied in protein isolates at different pH values suggests that UST at this point showed a favorable effect on improving the solubility for both isolates, as a result of the conformational changes during the UST that facilitated the formation of soluble aggregates increasing the protein-water interactions principally by unfolding the protein and reducing the particle size. These data were consistent with previous studies, finding that UST can cause an increase in protein solubility in different extraction media or buffers (Jambrak et al. 2009).

### Free sulfhydryl groups

**Fig. 5:**
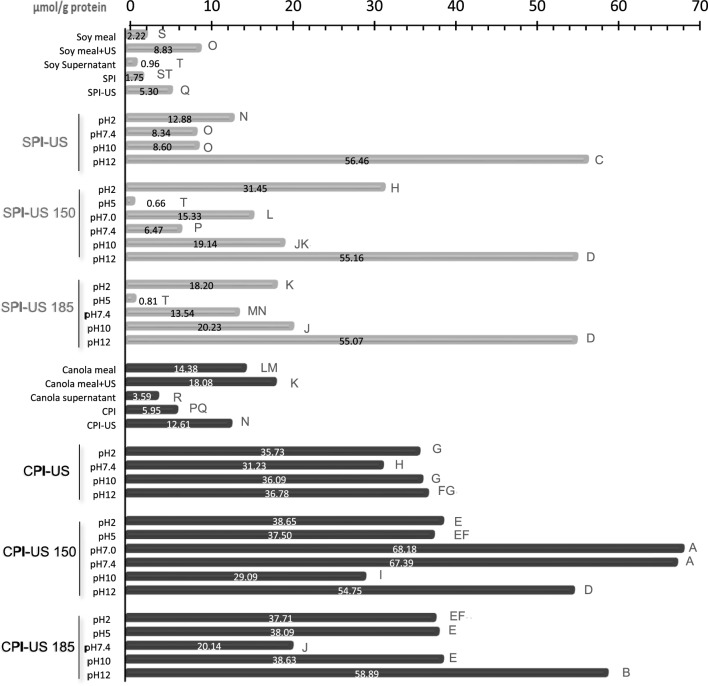
Free sulfhydryl groups determined in canola (CPI) and soybean (SPI) protein isolates obtained with conventional method with ultrasound-assisted extraction (150 kJ/L, 100% amplitude, USe2) (CPI-US, SPI-US). Also included protein isolates treated with additional ultrasound of 185 or 150 kJ/L (CPI-US 185, SPI-US 185; CPI-US 150, SPI-US 150, respectively). The meals and protein isolates were dispersed with water and phosphate buffer to obtain different pH values

Soybean meal showed lower $SH$ content than canola meal. An increase in $SH$ was observed in protein isolates (Fig. 5), due to the cavitation and mechanical forces generated during the UST causing protein unfolding and increased exposure of $SH$ to the surface of the protein, according to results reported by Hu et al. (2013). Furthermore, the increase was associated with the cleavage of the disulfide bonds that promoted their decrease to form new $SH$ groups (Wu et al. 2019). The highest $SH$ content was obtained at pH 12 in SPI without significant differences due to applying an additional UST. In CPI-US, the highest $SH$ content was also obtained at pH 12. The additional UST for CPI-US (150 or 180 kJ/L) did affect the $SH$ content at neutral pH. For soybean meal and SPI, the increase in $SH$ could occur on the basic polypeptides of glycinin (11S) containing at least 20 disulfide groups. Glycinin subunits are each linked by disulfide bonds, which can correlate to the increase in $SH$ due to unfolding of the protein with UST at different pH values. Canola proteins are composed of the 12S globulin (cruciferin) and 2S albumin (napin) forms, the latter is a small, basic, water-soluble protein held together by two disulfide bonds, that have a crucial role in the increase in $SH$ (Pan et al. 2020). Furthermore, it was observed that the higher the SH, the higher the solubility, which largely depends on the ${E}_{density}$ and the pH of the sample.

## Conclusion

The proposed ultrasound process as an environmentally friendly method, consumes less energy and has less processing time, therefore represents an alternative to recover and modify the physicochemical properties of proteins obtained from industrial byproducts. The recovery and protein content, regardless of the culture and preprocessing conditions, depended especially on the solvent:meal ratio and the ${E}_{density}$ applied. The alkaline extraction conducted in this study (pH 12 and 9.5 for canola and soybean, respectively), modified the surface charge of the proteins, improving their solubility in water.

The canola byproduct requires prior extraction to reduce antinutrients to contribute to its functionality, whereas the UST with 80% ethanol was the most effective for extracting phenolic compounds with less toxic solvents, less loss of soluble protein, and less extraction time. Furthermore, UST improved the solubility of proteins, and disrupted disulfide bonds, increasing the content of $SH$. In addition, ${E}_{density}$ used allows the extraction process to be scaled to an industrial level.

## Supplementary Information

Below is the link to the electronic supplementary material.

Supplementary file1 (DOCX 1260 kb)

## Data Availability

Data will be made available on request.

## Abbreviations

BSA: Bovine serum albumin
CM: Conventional method
CPI: Canola protein isolate
CPI-US: Canola protein isolate with UST
DTNB: 5,5′-Dithiobis(2-nitrobenzoic acid)
${E}_{density}$: Ultrasound energy density
GAE: Gallic acid equivalent
$PRY$: Protein recovery yield
$$PS$$: Protein solubility
$SH$: Free sulfhydryl groups
SPI: Soybean protein isolate
SPI-US: Soybean protein isolate with UST
T_room_: At room temperature ($\sim$ 21 °C)
UST: Ultrasound treatment
