# Supplementary material for: Ultrasound-assisted process to improve proteins recovery from industrial canola and soybean byproducts
Source: J Food Sci Technol. 2024 Oct 14;62(7):1350–61. doi: 10.1007/s13197-024-06108-8 (PMC12174011; doi:10.1007/s13197-024-06108-8)
Supplement: Supplementary file 1 — Supplementary file1 (DOCX 1260 kb) [file 13197_2024_6108_MOESM1_ESM.docx]

Table S1. Phenolic compounds evaluated as gallic acid equivalent (GAE), extracted in sonicated canola meal and in protein isolates obtained by conventional method, solvents are indicated.

| Polyphenol content  (mg GAE/100 g) | | | |
| --- | --- | --- | --- |
| Canola meal  (104 kJ/L)  water | Canola meal  (104 kJ/L)  methanol:ethanol:water  (7:7:6 v/v) | Canola meal  (104 kJ/L)  acetone:water  (7:3 v/v) | Canola meal  (104 kJ/L)  ethanol:water  (8:2 v/v) |
| 5558.5 ± 12.3 | 3860.6 ± 9.0 | 4212.2 ± 76.7 | 4029.1 ± 16.0 |
|  |  |  |  |
| Canola protein isolate  water | Canola protein isolate  ethanol:water  (8:2 v/v) | Soy protein isolate  water | Soy protein isolate  ethanol:water  (8:2 v/v) |
| 74.4 ± 2.7 | 357.3 ± 8.34 | 40.7 ± 2.9 | 95.2 ± 0.49 |

Mean ± standard deviation

Table S2. Chemical composition of supernatants.

|  | Soy supernatant | Canola supernatant |
| --- | --- | --- |
| Moisture (%) | 96.47$\pm$0.4^A^ | 97.47$\pm$0.4^B^ |
| Soluble proteins (%) | 3.82 $\pm$0.014^C^ | 3.79 $\pm$0.22^C^ |
| Soluble carbohydrates (%) | 0.504 $\pm$ .01^D^ | 0.327$\pm$0.03^E^ |
| Polyphenol content (mg GAE/100 g) | 0.14 $\pm$0.001^F^ | 0.18 $\pm$0.004^F^ |

Mean ± standard deviation. Different letters (A, B, C) show statistically significant differences between samples with a 95% confidence level.


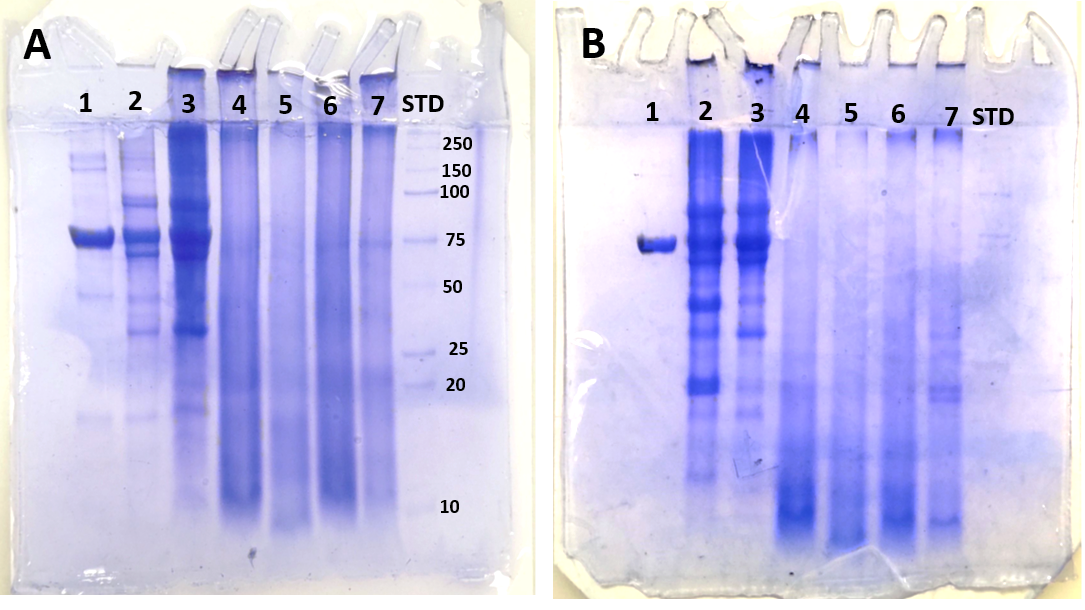


Fig. S1. Electrophoresis SDS-PAGE: A) nonreduced conditions, B) reduced conditions. Lane 1: bovine serum albumin, Lane 2: soybean protein isolate, Lane 3: soybean protein isolate + ultrasound (150 kJ/L), Lane 4: canola protein isolate at pH at 7.4, Lane 5: canola protein isolate at pH 8.0, Lane 6: canola protein isolate at pH 10, Lane 7: Canola protein isolate + ultrasound (150 kJ/L), STD: standard.
